# Molecular Approaches Detect Early Signals of Programmed Cell Death in *Hippolyte inermis* Leach

**DOI:** 10.3390/cimb46060368

**Published:** 2024-06-18

**Authors:** Francesca Glaviano, Roberta Esposito, Emanuele Somma, Amir Sagi, Eliahu D. Aflalo, Maria Costantini, Valerio Zupo

**Affiliations:** 1 Department of Integrative Marine Ecology, Ischia Marine Centre, Stazione Zoologica Anton Dohrn, 80077 Ischia, Italy; francesca.glaviano@gmail.com (F.G.); emanuele.somma@szn.it (E.S.); 2 Department of Ecosustainable Marine Biotechnology, Stazione Zoologica Anton Dohrn, Via Ammiraglio Ferdinando Acton n. 55, 80133 Naples, Italy; roberta.esposito@szn.it; 3 Department of Life Science, University of Trieste, Via L. Giorgieri, 10, 34127 Trieste, Italy; 4 Department of Life Sciences, Ben-Gurion University of the Negev, P.O. Box 653, Beer-Sheva 8410501, Israel; sagia@bgu.ac.il (A.S.); aflaloe@bgu.ac.il (E.D.A.); 5 Department of Life Sciences, Achva Academic College, Arugot 7980400, Israel

**Keywords:** aquaculture, gene expression, marine biotechnology, sex reversal, shrimp

## Abstract

The protandric shrimp *Hippolyte inermis* is the only known marine invertebrate whose sex determination is strongly influenced by the composition of its food. In *H. inermis*, a sex reversal is triggered by the ingestion of diatoms of the genus *Cocconeis* associated with leaves of the seagrass *Posidonia oceanica*. These diatoms contain compounds that promote programmed cell death (PCD) in *H. inermis* and also in human cancer cells. Transcriptomic analyses suggested that ferroptosis is the primary trigger of the shrimp’s sex reversal, leading to the rapid destruction of the androgen gland (AG) followed by a chain of apoptotic events transforming the testes into ovaries. Here, we propose a molecular approach to detect the effects of compounds stimulating the PCD. An RNA extraction method, suitable for young shrimp post-larvae (five days after metamorphosis; PL_5_ stage), was established. In addition, six genes involved in apoptosis, four involved in ferroptosis, and seven involved in the AG switch were mined from the transcriptome, and their expression levels were followed using real-time qPCR in PL_5_ fed on *Cocconeis* spp., compared to PL_5_ fed on a basic control feed. Our molecular approach, which detected early signals of sex reversal, represents a powerful instrument for investigating physiological progression and patterns of PCD in marine invertebrates. It exemplifies the physiological changes that may start a few days after the settlement of post-larvae and determine the life destiny of an individual.

## 1. Introduction

Model organisms permit us to investigate biological processes [1] in several research areas, including ecotoxicology, molecular biology, and evolutionary developmental biology, and may be leveraged to provide data for biotechnological applications [2,3]. As a result, specific tools and detailed protocols have become widely available in the literature [4]. In this context, crustaceans are often employed as models in the study of sexual differentiation and development by virtue of the functions of their androgenic gland (AG), a unique crustacean endocrine organ that secretes an insulin-like androgenic hormone (IAG) [5,6], which acts as a ‘switch’ for sexual development [7,8,9,10,11,12]. In fact, the simple presence of an AG triggers the development of males, while the absence of this gland (or its destruction in protandric species) triggers the development of females. Importantly, despite the need for laborious maintenance in the laboratory, some species are used as models because they are the only known targets for specific compounds, or because they may be used to investigate peculiar physiological pathways [13,14]. One such species is *Hippolyte inermis* Leach, a protandric shrimp living mainly on the leaves of the Mediterranean seagrass *Posidonia oceanica* [15]. We note that although the sex differentiation of crustaceans has been investigated in many species [16,17,18], *H. inermis* represents a peculiar case [19,20] because it undergoes a process of sex reversal triggered by the ingestion of diatoms [21]. A bioactive compound produced by diatoms upon wounding [22] triggers early development of *H. inermis* towards femaleness, as a result of coevolutionary processes within the leaf stratum of *P. oceanica* and other seagrasses [21,23]. *H. inermis* is characterized by two reproductive bursts [22], one taking place in the spring, and the other in the fall. In the spring, both males and females are born, while the fall season produces only males, which undergo sex reversal after about one year [23]. The presence of young females in spring is due to an early sex reversal triggered by the ingestion of diatoms of the genus *Cocconeis* [21], which are particularly abundant in that season [23]. This change is a consequence of a programmed cell-death (PCD) trigger directed selectively towards AG cells [24,25]. The chemical structure of the bioactive compound is still under investigation, but it is known to be a small lipophilic compound [26], probably similar to dihomo-γ-linolenic acid (DGLA) [27].

Bioassays using *H. inermis* require complex culture practices [28], because the sex ratio of animals fed on diatom fractions is evaluated 50–60 days after hatching, when it is possible to detect the activity of cell-death compounds. This procedure requires the sacrifice of mature shrimps and their analysis under a stereomicroscope to check for the presence of an appendix masculina on the second pleopod [18,22,29]. Molecular tools for *H. inermis* are, at present, very scarce, but Levy et al. [30] have obtained transcriptomic libraries of representative development stages (immature, male, young female and mature female). However, previous studies [27] revealed a set of genes influenced by the ingestion of diatoms, demonstrating the remarkable role of ferroptosis as a form of PCD specifically acting on still undifferentiated gland tissues. This mechanism appears to be a smart physiologic strategy to produce massive changes in the physiology of an invertebrate by targeting a few young cells, and it has been demonstrated to be evolutionarily conserved, from *Caenorabditis elegans* to humans [27] and, of course, is present in the model shrimp *H. inermis*. In this crustacean, several genes are activated by the ingestion of diatoms, and they are involved in ferroptosis of the AG, the apoptosis of the testes, and the activation of the IAG. Remarkably, diatom ingestion triggers most physiological changes in young post-larvae just 5 days post-metamorphosis (PL5). In particular, it was demonstrated [27] that the whole process of sex reversal in this protandric species is completed within the first 2 weeks of post-larval development and that 5 days after the metamorphosis was the optimal stage at which to detect genic changes involved in the destruction of the AG and the consequent start of the ovary development, upon the feeding on diatoms. In the present experimental work, aimed at investigating the effects of natural compounds triggering PCD in selected shrimp tissues, an RNA extraction method was developed for the PL_5_ stage. Furthermore, several genes involved in apoptosis, ferroptosis, and insulin-like secretion, previously demonstrated to be involved in the sex reversal of *H. inermis*, were isolated from the transcriptome. Their expression levels were followed by real-time qPCR in PL_5_ fed on *Cocconeis* spp., as compared to PL_5_ fed on a basic feed (without diatoms). These data were compared with those obtained from the transcriptome [27] to investigate the physiological responses ruled by PCD and their functional relationships by means of interactomic analyses.

## 2. Materials and Methods

### 2.1. Sample Collection and Culture Practices

**Figure 1:**
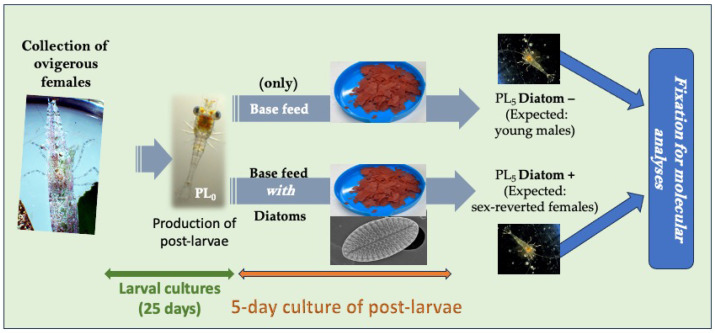
Culture of shrimps for molecular analyses. Three replicates of negative controls (designated Diatom −) were fed for 5 days on a basic feed (not containing diatoms). Three replicate treatments (designated Diatom +) were fed for 5 days on the same basic feed with the addition of diatoms. Post-larvae were collected after 5 days and fixed in RNA stabilization reagent for molecular analyses.

Ovigerous females of *H. inermis* were collected at Lacco Ameno, Ischia (Bay of Naples, Italy; 40°44′56″ N, 13°53′13″ E) in a *P. oceanica* meadow. Samples were screened under a Leica MZ6 stereomicroscope (Leica Microsystems, Milan, Italy) to confirm the taxonomical identification of shrimps, based on their morphological characters [23]. *H. inermis* ovigerous females were individually reared in a thermostatic chamber (18 °C) in aerated 2 L conical flasks filled with filtered (0.45 µm) seawater, until the release of larvae. Larvae were collected with Pasteur pipettes, pooled in groups of 80 individuals, and transferred to 1 L conical flasks containing 800 mL of filtered (0.45 µm) seawater (1 larva per 10 mL of seawater). Larval cultures were maintained in the same thermostatic chamber (18 °C) for 25 days (Figure 1). During this period, the seawater in each conical flask was changed every other day. Larvae were fed on *Artemia salina* (4 nauplii per mL) and *Brachionus plicatilis* (4 individuals per mL) up to their settlement, and larval foods were replaced every other day. After the settlement, the post larvae were transferred to 500 mL crystallization dishes in groups of 25 individuals and shifted onto the experimental diets. Following the method proposed by Zupo et al. [27], this phase lasted for 5 days. Post-larvae used as negative controls (designated Diatom –) were fed on a basic feed composed of dried *A. salina*, SHG Hi-Red, dried *Spirulina*, and SHG Microperle in equal amounts (Figure 1). Post-larvae used for the treatment (designated Diatom +) were fed on the basic feed enriched with 30% (by weight) lyophilized *Cocconeis scutellum* var. *parva* [25]. Five days after settlement (PL_5_ stage), 75 post-larvae deriving from 3 treatment and 3 control replicates (Table 1) were sacrificed and individually fixed in RNAlater (RNA Stabilization Reagent, Qiagen, Hilden, Germany).

In parallel, other settled post-larvae deriving from the larval cultures were transferred in pools of 25 individuals into three replicates in 500 mL crystallization dishes for the control diet (Diatom −) and 3 replicates for the treatment diet (Diatom +). In this case, however, the post-larvae were cultured for 45 days, up to complete sex maturation (Table 1). At the end of the experiment, adult shrimps (having a total length greater than 7.0 mm) were fixed in 70% ethanol and further examined to determine their sex. Sex analysis was performed under a Leica MZ6 stereomicroscope (Leica Microsystems, Milan, Italy) by ablating the second pleopod, which was examined on a freshly prepared slide, under a Leica DMLB optical microscope (Leica Microsystems, Milan, Italy). The presence of an appendix *masculina* [31] indicates a male animal [23]. To validate the transcriptomic results, further analyses performed on the PL_5_ consisted of monitoring the expression of genes in males, deriving them from negative controls (Diatom −), and in females, deriving them from treatments with feed containing diatoms (Diatom +). The data collected on the sex ratios in each replicate were expressed as the number of females compared to the total number of mature individuals (F/mat%; [9]), for both control and treated individuals.

### 2.2. RNA Extraction and cDNA Synthesis

Two methods of RNA extraction were compared, and they were both tested on a variable number of individuals (viz, 1, 2, 3, and 4 PL_5_, respectively). The first method employed the RNeasy Mini Kit. In this case, post-larvae were lysed using a variable quantity of RLT/2-ME buffer (10 μL β-mercaptoethanol for each mL of RLT buffer), according to the number of PL_5_: 350 μL for 1 PL, 400 μL for 2 PL, 450 μL for 3 PL, and 600 μL for 5 PL. Samples were homogenized with TissueLyser (Qiagen, Austin, TX, USA), using 3 mm sterile aluminium beads at 20.1 Hz for 3 min. RNA was extracted following the manufacturer protocol (Qiagen, Austin, TX, USA) and eluted with 30 μL RNase-free water, then stored at −80 °C. The second method employed a PureLink™ RNA Mini Kit. Post-larvae were lysed in lysis buffer (containing β–mercaptoethanol, according to the number of PL_5_: 350 μL for 1 PL, 400 μL for 2 PL, 450 μL for 3 PL, and 600 μL for 5 PL) in TissueLyser (Qiagen, USA) using 3 mm sterile aluminium beads at 20 Hz for 3 min. RNA was extracted according to the manufacturer’s protocol (Thermo Fisher Scientific, Waltham, MA, USA). RNA was eluted with 30 μL of RNase-free water provided by the kit. The samples obtained were then stored at −80 °C.

The quality of total RNA extracted using both methods was estimated by Nanodrop (ND-1000 UV Vis, NanoDrop Technologies, Wilmington, DE, USA), measuring the absorbance at 260 nm and 260/230 and 260/280 nm ratios, to exclude the presence of proteins, phenols, and other contaminants. The integrity of the RNA was finally assessed by running 100–200 ng of RNA samples on 0.8% agarose gel. For each sample, 600 ng of total RNA extracted was retrotranscribed with an iScript cDNA synthesis kit (Bio-Rad, Milan, Italy), according to the manufacturer’s instructions. In terms of the amount of total RNA extracted, we found that the RNeasy kit was the most efficient method, with respect to the PureLink™ RNA Mini Kit, and it was used to analyze both the Diatom + and Diatom − groups.

### 2.3. Identification of Genes

The sequences of 17 genes belonging to 3 specific molecular pathways, i.e., ferroptosis, apoptosis, and insulin-like secretion, were previously detected in the transcriptome of *H. inermis* [29,30] (see Table 2 and Table S1 for their functions). They were selected according to Zupo et al. [27] along with the sequences of two housekeeping genes, *cytochrome oxidase subunit* (*COI*) and *18S ribosomal RNA*. For each gene, specific primers were designed on the basis of nucleotide sequences and used to amplify the selected fragments with a Taq High-Fidelity PCR System (Roche, Monza, Italy). The amplification reactions were performed in 30 μL final volume with 3 μL of 10× PCR reaction buffer, 3 μL of 10× 2 mM dNTP, 1 μL of 5 U/μL Taq, and 100 ng/μL of each primer, template cDNA, and nuclease-free water. The PCR program consisted of a cDNA denaturation step at 95 °C for 5 min, 35 cycles at 95 °C for 45 s, 54–60 °C for 1 min, and 72 °C for 30 s, and then a final extension step at 72 °C for 10 min. The fragments were further purified from agarose gel using the QIAquick Gel Extraction kit (Qiagen, Milan, Italy), and their specificity was checked by DNA sequencing. PCR products were finally aligned with gene sequences by means of MultAlin (available at http://multalin.toulouse.inra.fr/multalin/).

### 2.4. Gene Expression by Real-Time qPCR

The specificity of the amplification reactions for each pair of primers was verified by melting curve analysis. The theoretical efficiency (E) of each primer pair was calculated according to a standard function, as follows:

E = 10^−1/slope^ (1)

where slope = the slope of the standard curve, plotted with the y axis as Ct and the x axis as log(quantity).

Five serial dilutions were prepared to generate standard curves, and Ct values were determined for each dilution by plotting Ct values against the logarithm of the corresponding dilution factor. PCR efficiencies were then calculated separately for both the control and target genes. PCR efficiencies were found to be high, indicating successful amplification of the desired gene fragments. Diluted cDNA was used as a template in a reaction containing a final concentration of 0.3 mM for each primer and 1× FastStart SYBR Green master mix (total volume 10 μL; Applied Biosystems, Monza, Italy). The following thermal profile was used: one cycle of 95 °C (10 min) for the cDNA denaturation; forty cycles of 95 °C (15 s) and 60 °C (1 min) for the amplification; one cycle of 72 °C (5 min) for the final elongation; and one cycle from 60 to 95 °C for melting curve analysis to verify the presence of a single product. Each RT-qPCR reaction was performed in three duplicates. Fluorescence was determined using ViiA^TM^7 software V1.3. The relative expression ratios were calculated from quantification cycles. Undiluted cDNA (1:1) was then chosen as a template to compare the expression of the genes of interest in samples obtained from individuals in the treatment and control groups. Three RNA replicates were used to synthesize cDNA for both the treatment and control groups, according to the protocol reported in Section 2.2. The relative expression ratios were calculated by the relative expression method, using REST software v2, a mathematical model based on the correction for exact PCR efficiencies and the mean crossing point deviation between sample groups and control groups. Differences higher than 2 were considered significant. The results were further compared with the findings obtained in the previous transcriptomic samples used by Levy et al. [30], aiming at ensuring the consistency and reliability of the gene expression data obtained in this investigation.

### 2.5. Interactomic Analysis

Network analysis was performed by Ingenuity Pathway Analysis Version 7.1 (IPA, Ingenuity Systems, Inc., Redwood City, CA, USA), based on associated functions and data mining from experimental studies previously reported [32] to identify the relationships between the genes analyzed in this study. The graphical representations display nodes (genes) and edges (the biological relationships between nodes). Since *H. inermis* genes are not annotated in the IPA database, we used the orthologous human genes to search for the genes of interest (Table 3).

### 2.6. Statistical Treatment of the Data

The quantitative differences between samples of total RNA obtained by the two RNA extraction methods were evaluated using a paired Student’s *t*-test; *p* values lower than 0.05 were considered significant. The qualitative differences between samples of total RNA obtained by the different RNA extraction methods were evaluated by comparing the A260/230 ratios, also using a paired Student’s *t*-test. The differences in the sex ratio between adult shrimps in three control replicates (Diatom −) vs. three replicates of the treatment (Diatom +) fixed at 45 days were determined by comparing the F/mat% (percentages of females out of the total of mature individuals) by means of a *Z*-test on proportions. Statistical analyses were performed using GraphPad PRISM (GraphPad Prism version 7.0.0 for Windows, GraphPad Software, San Diego, CA, USA, www.graphpad.com; 15 December 2023).

## 3. Results

### 3.1. Analysis of Sex Ratios

**Figure 2:**
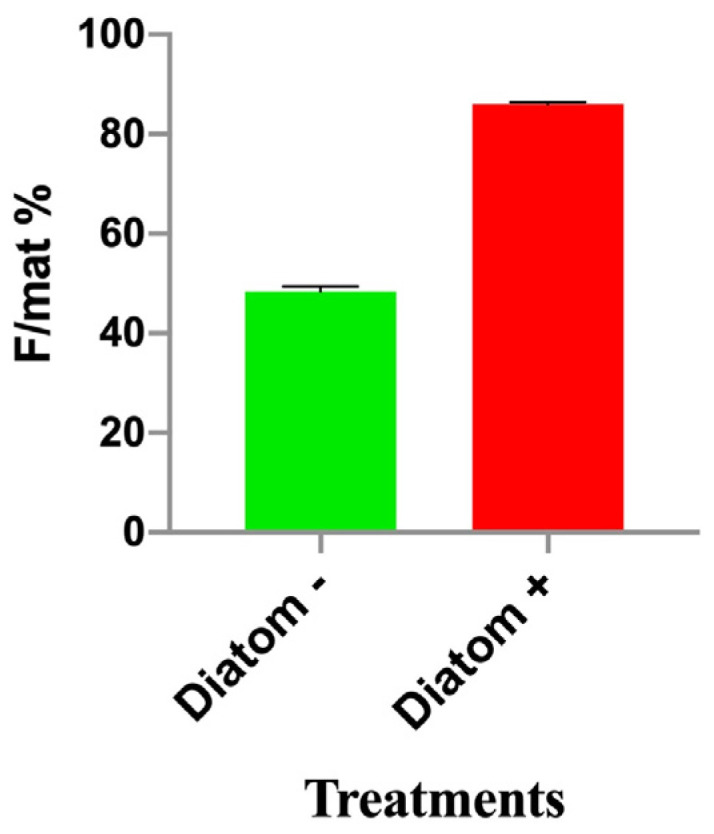
Percentage of females out of the total number of mature individuals (F/mat%) obtained in groups raised on feed without diatoms (Diatom −) vs. feed containing diatoms (Diatom +).

The percentage of females out of the total number of mature individuals in the experimental groups fed for 45 days on basic feed (Diatom −) was 48.36% (±1.06), while that for the treated groups (Diatom +; Figure 2) was 86.06% (±0.32). The differences between the two experimental groups were significant (*z*-test on proportions, *p* < 0.001), and this result demonstrated that the feeding on diatoms triggered the treated shrimps’ development into females.

### 3.2. Evaluation of RNA Quantity and Quality

Different results were achieved using the two extraction kits. The number of PL_5_ pooled and extracted also influenced the quality of extracted RNA. A significantly higher quantity of total RNA was extracted (paired *t* test; *p* = 0.038) using the RNeasy kit, and this result was consistent in all analyzed samples (Table 4). A significant difference was also found in the RNA purity, according to the A260/230 ratio (paired *t* test; *p* = 0.014), although the difference for the A260/280 ratio was not significant. Taking into account that the minimum amount of total RNA required for cDNA synthesis is ~30 ng/μL according to the iScript™ cDNA Synthesis kit (Bio-Rad), the RNeasy kit was the most efficient method for the production of RNA for our analyses.

### 3.3. Gene Expression

**Figure 3:**
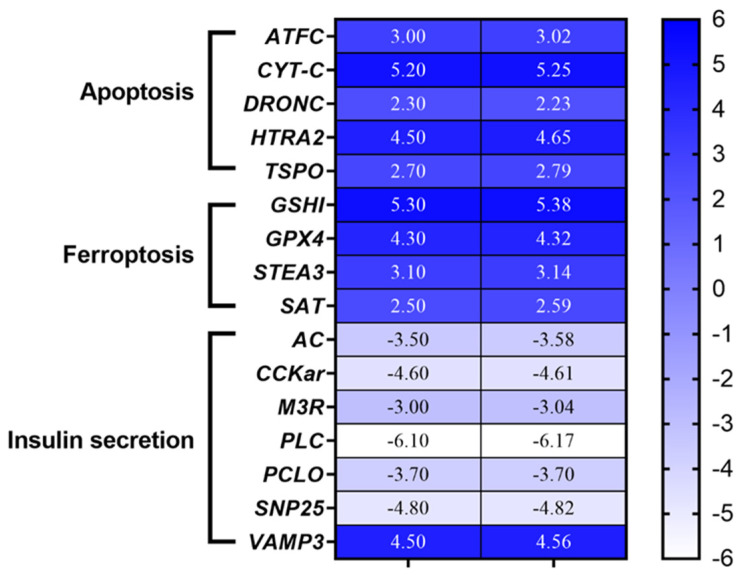
Heatmap showing the expression profiles of downregulated and upregulated genes analyzed by real-time qPCR, derived from the transcriptome (left) and from the newly tested samples (right). Genes of 5-day-old (PL_5_) *H. inermis* fed on basic feed plus lyophilized *Cocconeis* spp. diatoms (Diatom + treatment) were compared with those of PL_5_ fed only on basic feed (Diatom − treatment) and their upregulation (darker color) or downregulation (lighter color) are shown according to the scale bar.

Expression levels of the genes of interest in PL_5_ of *H. inermis* fed on lyophilized *C. scutellum* var. *parva* (Diatom +) were compared with those for the PL_5_ fed on the basic feed (Diatom −), as was previously performed for the transcriptomic analyses reported by Levy et al. [30]. The expression levels of each gene were analyzed and internally normalized against the negative control (Diatom −) and then further compared with the transcriptome data (Figure 3).

All genes involved in the apoptosis pathway were significantly upregulated in Diatom+ treatments, in accordance with the levels of gene expression detected by the transcriptome (Figure 3). The genes involved in the insulin-like secretion pathway were all downregulated in the treated individuals (Diatom +), as compared to controls (Diatom −), except for Vamp 3, which was upregulated. Another key process that showed an alteration in gene expression triggered by the ingestion of *Cocconeis* diatoms was cell death by ferroptosis. Consequently, the results obtained for the ferroptosis pathway in the samples here processed were consistent with the conclusions based on the transcriptome (Figure 3). The differences between the samples processed here (Figure 3, right) and the transcriptome (Figure 3, left) were not significant, according to the Student’s *t* test (*p* < 0.01).

### 3.4. Network Analysis

**Figure 4:**
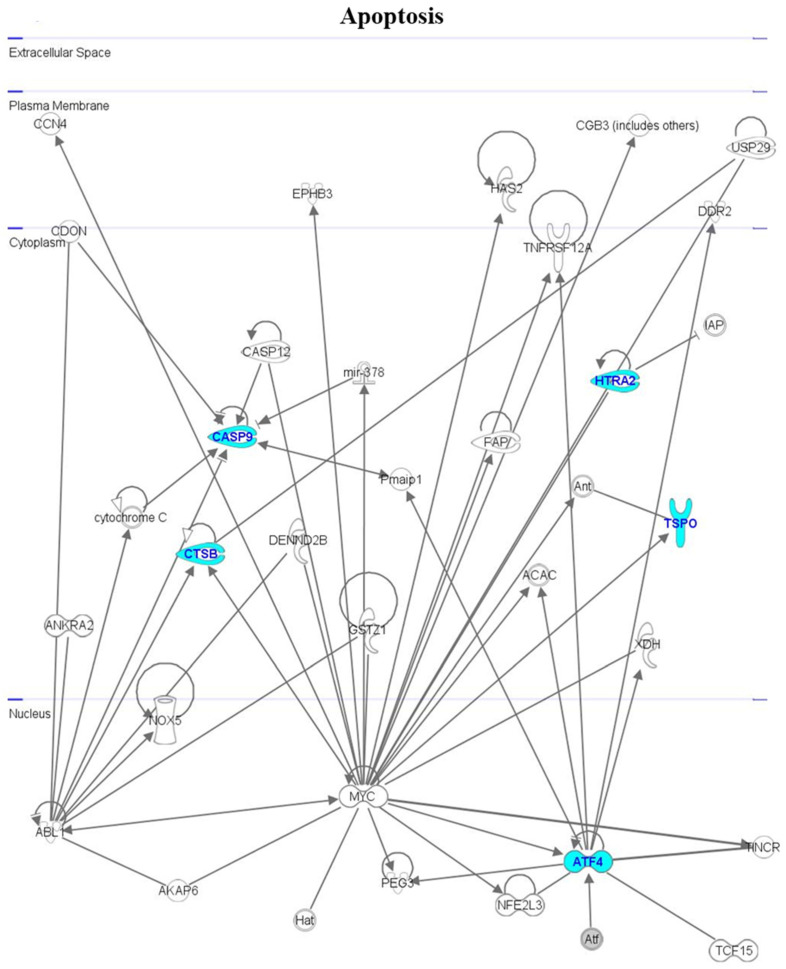
Interactomic analysis by the Ingenuity Pathway Analysis (IPA) software (version 7.1), showing the network of genes involved in apoptosis. The genes that were analyzed are shown in blue. The biological relationships between the significant genes are indicated by arrows (indicating that one molecule modulates the expression of another). The connections indicated by edges (and not by arrows) indicate direct relationships between molecules due to real chemical modifications and, hence, to the formation of direct physical contacts. For further details of IPA analysis, see Section 2 (paragraph 2.5).

Interactomic analysis indicated that all the genes analyzed in this study exhibited a large degree of interaction, and that they have connections with many other genes. For the genes involved in the apoptosis pathway, the following interactions were revealed (Figure 4): *ATFC* interacts with *Nuclear factor-erythroid 2 P45-related factor 3* (*NFE2L3*), *Paternally-expressed gene 3 protein* (*PEG3*), *Terminal differentiation-induced NcRNA* (*TINCR*), and *Transcription factor 15* (*TCF15*); *CTSB* interacts with *ABL proto-oncogene 1* (*ABL1*), *Proto-oncogene C-myc* (*MYC*), and *TNF receptor superfamily member 12A* (*TNFRSF12A*); *Cyt-c* interacts with *CASP9* and *ABL1*; *CASP9* interacts with *Cytochrome C*, *ABL1*, *Cell adhesion associated*, *Oncogene regulated* (*CDON*), *Caspase 12* (*CASP12*), and *Phorbol-12-myristate-13-acetate-induced protein 1* (*Pmaip*); *HTRA2* interacts with *MYC*, *inhibitor of apoptosis* (*IAP*), and *Discoidin domain receptor tyrosine kinase 2* (*DDR2*); and *TSPO* interacts with *Adenine nucleotide translocase* (*Ant*) and *MYC*.

**Figure 5:**
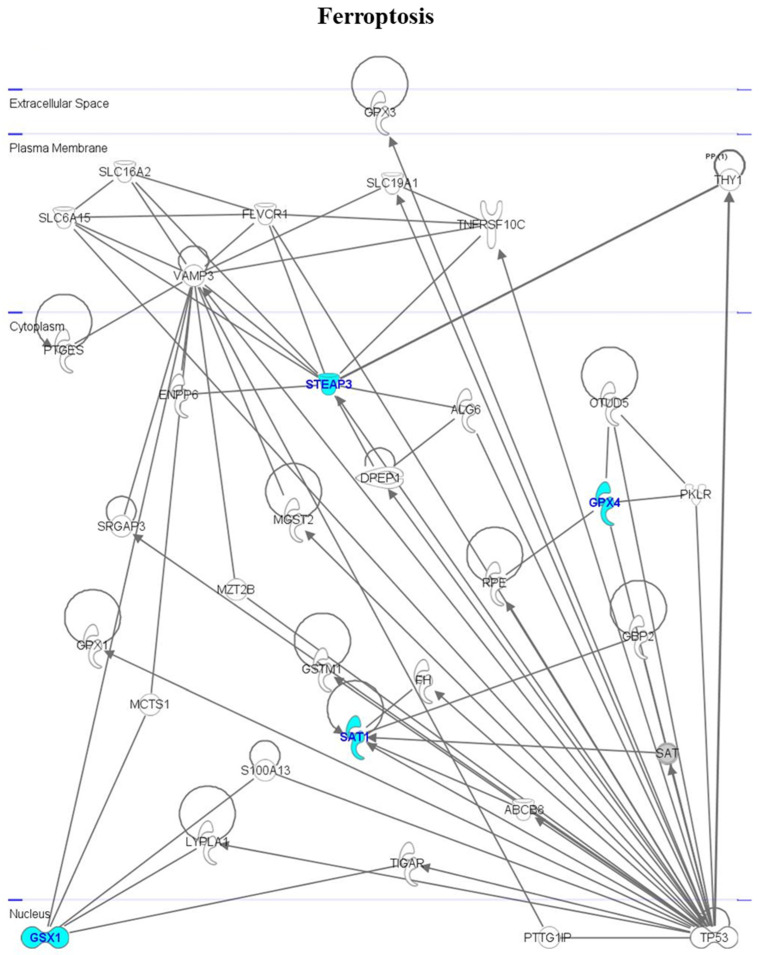
Interactomic analysis by Ingenuity Pathway Analysis (IPA) software, showing the network of genes involved in ferroptosis. For further details, see legend to Figure 4.

Genes involved in ferroptosis are functionally intercorrelated as follows (Figure 5): *GSX1* interacts with *VAMP3*, *malignant T-cell amplified sequence 1* (*MCTS1*), *S100 calcium binding protein A13* (*S100A13*), *Lysophospholipase 1* (*LYPLA1*), and *TP53-induced glycolysis regulatory phosphatase* (*TIGAR*); *GPX4* interacts with *Pyruvate kinase L/R* (*PKLR*), *OTU deubiquitinase 5* (*OTUD5*), *ribulose-5-phosphate-3-epimerase* (*RPE*), *glutathione peroxidase*, and *Tumor protein P53* (*TP53*); *STEAP3* interacts with *Solute carrier family 6 member 15* (*SLC6A15*), *VAMP3*, *Solute carrier family 16 member 2* (*SLC16A2*), *FLVCR heme transporter 1* (*FLVCR1*), *dipeptidase 1* (*DPEP1*), and *TP53*; and *SAT* interacts with *fumarate hydratase* (*FH*), *guanylate binding protein 2* (*GBP2*), *DPEP1*, and *TP53*.

**Figure 6:**
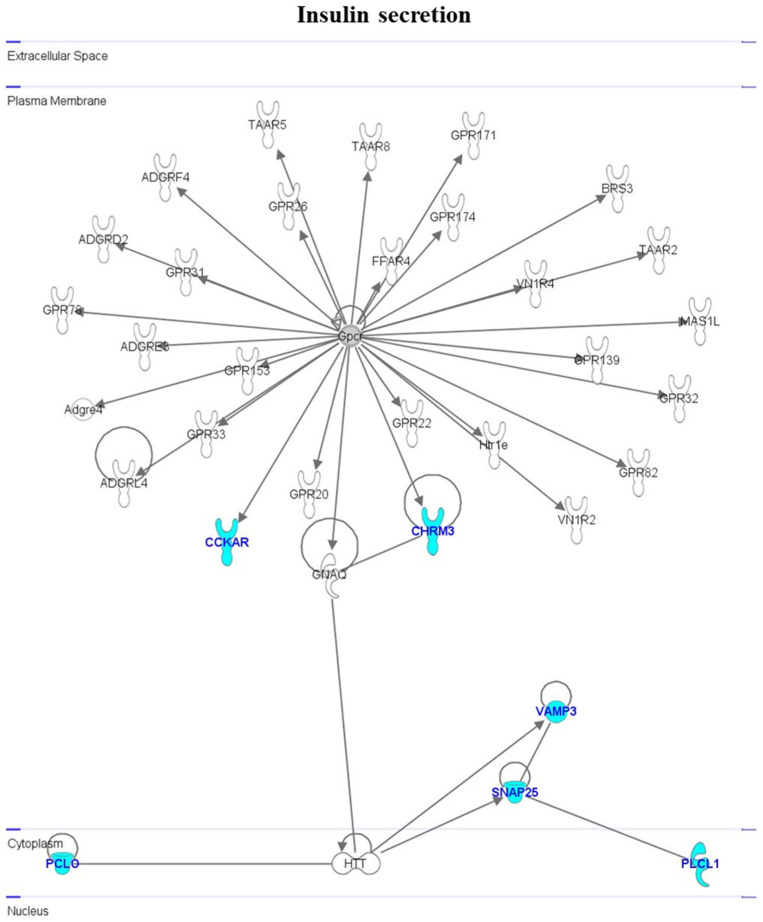
Interactomic analysis by Ingenuity Pathway Analysis (IPA) software, showing the network of genes involved in insulin-like secretion. For further details, see the legend to Figure 4.

The close functional association between genes involved in insulin-like secretion (Figure 6) may be summarized as follows: *CCKAR* interacts with *G protein-coupled receptor* (*Gpcr*); *CHRM3* interacts with *G protein subunit alpha Q* (*GNAQ*) and *Gpcr*; *PLCL1*, *SNAP25*, and *VAMP3* are tightly linked, one to the other, and also interact with *Huntington Disease Protein* (*HTT*); *PCLO* also interacts with *HTT*.

## 4. Discussion

The balance between cell division and cell death is of utmost importance for the development and maintenance of multicellular organisms, and several model organisms have been employed in the past few decades to investigate the phases and the mechanisms of apoptosis and other PCD pathways [13,33]. The shrimp *H. inermis* has garnered increasing interest [21] as an effective model to detect early processes of PCD promoted by bioactive molecules. In fact, the mechanism of disruption of the AG in *H. inermis*, triggered by the lipophilic compounds contained in benthic diatoms (on which these organisms feed), may be considered a consequence of coevolutionary processes [34] that are influenced by various environmental factors [35]. However, the bioassays routinely performed with this organism to investigate the role/s of algal compounds in PCD have only partially revealed the complexity of mechanisms involved in this peculiar process of sex reversal [23,36,37]. The results of this study confirm that the ingestion of diatoms triggers an early process of sex reversal in young post-larvae [27] and that a larger proportion of females in diatom-fed individuals corresponds to the activation of a complex gene network. Previous investigations [23] demonstrated that the proportion of females deriving from spring reproductive bursts is quite skewed, and laboratory experiments [21] indicated that the ingestion of diatoms leads to lower percentages of males (with variable sex ratios according to the culture conditions, always reaching about 20–45% of males on the total number of adults). In contrast, the fall reproductive burst (when *Cocconeis* spp. are almost absent) leads to proportions of females generally lower than 30–40%, leading to an opposite trend in the sex ratios [23]. Consequently, the distribution of sexes is always skewed, because variable percentages of shrimps (lower in fall, when *Cocconeis* spp. are less abundant, higher in spring, when those diatoms are abundant) are prematurely subjected to sex reversal.

However, culturing a large number of sensitive larvae and post-larvae may occasionally influence their health and stress status [38], leading to unclear results [39,40]. In contrast, the molecular mechanisms activated by the ingestion of diatoms in young post-larvae correspond to those previously identified [27] and include: (i) the activation of a ferroptotic PCD involving the whole androgenic gland, (ii) the consequent apoptotic death of the testes tissues, and (iii) the interruption of the production of insulin-like hormone (IAG switch) leading to the development of female sex. This demonstrates that molecular approaches may represent a valuable alternative to traditional bioassays, providing insights into the understanding of the cellular machineries underlying sex reversal [41]. Such molecular approaches revealed important cellular strategies of PCD, whose impairment may have pathologic consequences and/or may lead to compromised embryogenesis [42,43], neurodegenerative diseases, or even the development of cancer. While it has long been known that canonical regulatory pathways involving members of the Bcl-2 and caspase families were established to regulate developmental apoptosis in *C. elegans* and flies [33,44,45,46], new animal models now offer the opportunity to discover multiple mechanisms involved not only in regulating cell death during mammalian development but also in tissue homeostasis and pathological forms of tissue decay promoted by PCD [46]. The need to investigate alternative PCD mechanisms is forcing us to seek newer models [47], often involving complex breeding procedures and sensitive bioassay techniques [48].

Taking into account that several crustaceans are employed for evolutionary developmental studies because of their unique physiologic properties [49,50,51,52], the results obtained are suitable for application in investigations on other protandric species. For example, *Parhyale hawaiensis* is a well-known amphipod for which an extensive toolbox for genetic manipulation is available [53,54,55,56] and several pandalid and hippolytid decapods exhibit interesting patterns of sex reversal, in addition to the other families of crustaceans containing hermaphroditic species. However, despite the availability of several model organisms, genetic variations of key cellular processes and their functional consequences have received less attention [16]. Many studies have been conducted on various caspase-dependent PCDs (involving apoptosis), but, to date, far fewer studies have been devoted to caspase-independent, non-apoptotic types of PCD [33,57,58] that play fundamental roles in the physiology of plants and animals [59]. Consequently, our study has contributed to revealing conserved molecules and phenomena that are less prominent in other classical model organisms. In particular, we may now explain how a peculiar ecological pathway—the sex reversal of a benthic shrimp used to stabilize its natural populations [23]—is triggered by a conserved mechanism of cell death [27]. In the case studied here, a clear example of environmental sex determination (ESD) is explained by the expression of a few genes, sequentially activated by the ingestion of a diatom. Since sex ratios are crucial for the shrimp population viability, it is evident that the spring blooms of *Cocconeis* spp. in their environment influence the sex ratio and interact with the genotypic sex determination (GSD), by anticipating the sex reversal (due to the early destruction of the AG) which in the fall generation takes place in shrimps aged about 1 year.

The genes under consideration were isolated for the first time from the transcriptome of *H. inermis*; they were chosen because they showed significant variations in expression following the ingestion of diatoms by this model organism and were, consequently, believed to play key roles in three major pathways involved in the *H. inermis* early sex reversal [6,46]. Functional analyses performed on these fundamental cellular processes revealed that these genes are functionally intercorrelated and also interact with other important gene networks [60]. A closer look at the above three pathways reveals that an apoptogenic mechanism is responsible for the premature destruction of the AG of *H. inermis* [23] as a selective mechanism of PCD. It is also known that an insulin-like secretion pathway constitutes a fundamental step in the regulation of the IAG switch produced by the AG [61] to control the sexual differentiation of decapod crustaceans [62]. Finally, it is worth noting that ferroptosis was previously shown to take place in *C. elegans* [63] and is considered crucial to promote additional cell death events in vertebrates [27,39]. In this investigation, it was confirmed, in agreement with a recent study [27], that ferroptosis is a mechanism evolutionarily conserved from *C. elegans* to humans.

In conclusion, the results of this investigation confirmed the patterns of expression indicated by previous transcriptomic analyses [27] and provide a combination of tools for the study of PCD in crustaceans and other invertebrates. In addition to genes involved in ferroptosis (four upregulated genes were found in treated post-larvae, namely *GSHI*, *GPX4*, *STEA3*, and *SAT*), six genes in the apoptosis pathway (*ATFC*, *CATB*, *Cyt-c*, *Dronc*, *HTRA2*, and *TSPO*) and seven genes in the insulin-like secretion pathway (*AC*, *CCKAR*, *M3R*, *PLC*, *PCLO*, *SNP25*, and *VAMP3*) [64] were differentially expressed. The downregulation of genes involved in the insulin-like secretion pathway indicates that a PCD cascade effect primed the destruction of the AG and that this led to the inhibition of the production of the insulin-like hormone [6,35,65,66,67]. In fact, this androgenic hormone is normally secreted by the AG in males [68]. Importantly, this study shows that interactomic analyses permit us to track the activity of a cell-death compound in 5-day-old post-larvae; this will lead to a deeper understanding of its mechanism of action, enabling the knowledge to be applied for medical and ecological biotechnologies. Indeed, previous investigations demonstrated that crude extracts of these diatoms specifically activate, in vitro, a dose-dependent PCD in human cancer cells (BT20 breast carcinoma) but not in human normal lymphocytes [60] and this finding opens new opportunities to apply natural products from diatoms for devising new cancer therapies [69]. Further studies will take advantage of the tools described here, permitting us to test the effect of cell-death compounds on sensible targets (such as the AG tissues of the shrimp) in very early stages of development, with lower effort and a clearer comprehension of the mechanisms of action. The possibility to test the effect of candidate compounds able to selectively destroy specific tissues will be important for cancer research. In addition, the elucidation of the molecular mechanisms underlying the sex reversal of crustaceans will also be useful to produce new aquaculture biotechnologies for monosex cultures of decapods, because cell-death compounds will be applied to induce the chemical destruction of the AG in cultured crustaceans and simplify the production of all-female or all-male populations, respectively. On the whole, the determination of the genic networks involved in the complex and peculiar process of sex reversal in *H. inermis* will contribute to a better understanding of the plant-animal relationships driving important chemical ecology processes in benthic environments.

## Figures and Tables

**Table 1:** Summary of experimental design. The preliminary larval growth phase is reported in the first row, while two experimental trials are reported in the second and third rows, with all the technical details. In the last column, the purpose of each experiment is indicated, i.e., the production of post-larvae, the long-term (45 days) maturation of sex, and the (5-day) production of PL_5_ for molecular investigations.

Duration (Days)	Experimental Phase	Density	Feed	No. of Replicates	Container	No. of Individuals/ Replicate	Purpose
25	Larval rearing	1 larva/ 10 mL	*Artemia + Brachionus*	10	Conical flasks	80	Production of post-larvae
45	Post-larval rearing	1 post-larva/ 20 mL	Diatom + vs. Diatom −	3	Dishes	25	Identification of sex
5	Post-larval rearing	1 post-larva/ 20 mL	Diatom + vs. Diatom −	3	Dishes	25	Molecular analyses

**Table 2:** Nineteen genes isolated from the transcriptome of *Hippolyte inermis* and classified according to the pathways in which they are involved (with acronym, gene name, primer name and sequences, and lengths of amplified fragments).

Gene Type	Acronym	Gene	Primer	Sequence 5′>3′	Fragment Length (bp)
Housekeeping	*COI*	*Cytochrome oxidase subunit*	Coi_Hi_F1	CTGAAGAGGTATAGTAGGAAC	204
			Coi_Hi_R1	CTCGGTGCCCCTGACATAGC	
	*18S RNA*	*18S ribosomal RNA*	18S_Hi_F1	CATGCATGTGTCAGTACAGGC	204
			18S_Hi_R1	CTTATCATATGAGAATCCAACC	
Apoptosis	*ATFC*	*Activating transcription factor*	ATFC_Hi_F1	GGCTGGAGTTCTGACAGAGG	189
			ATFC_Hi_R1	CAGCCCAGCTCTTCCAGATTG	
	*CATB*	*Cathepsin B*	CATB_Hi_F2	GGATCTTGTGGATCATGCTGG	198
			CATB_HI_R2	GTTCCAGCCATCCGCCATTAC	
	*Cyt-c*	*Cytochrome C*	CYT_Hi_F1	GTGCAGAGATGTGCTCAGTGC	167
			CYT_Hi_R1	ACATCCAGAGTGTCATCTGC	
	*Dronc*	*Death receptor-associated nemesis-like*	DRONC_Hi_F1	GGCATCATTATGACAGATATGC	201
			DRONC_Hi_F1	GTGTGATGATATCATGTAGAGC	
	*HTRA2*	*High-temperature requirement A2 serine peptidase*	HTRA2_Hi_F2	GACACAATGAAGCCAGAGCC	190
			HTRA2_Hi_R2	CGCCATCAGTTCTCTGCTAG	
	*TSPO*	*Translocator protein*	TSPO_Hi_F1	GCAGGTGGCAAATGAAATGGAG	132
			TSPO_Hi_R1	CTGGCGTCCTCCTAACTGGATG	
Ferroptosis	*GSHI*	*Gamma glutamylcysteine synthetase*	GSH1_Hi_F1	GCCGTGTGAAGTCCAGCTGA	242
			GSH1_Hi_R1	CATTCACGGACATCTGACTAG	
	*GPX4*	*Glutathione peroxidase 4*	GPX4_Hi_F1	GCTGAGAGTCTGAGAGACTG	195
			GPX4_Hi_R1	CTAGTCACTAAACGTCGTCGG	
	*STEA3*	*Sterile alpha-motif domain-containing protein 3 metalloreductase*	STEA3_Hi_F1	GAGCATATGCAGATAACGTG	185
			STEA3_Hi_R1	GGCTATTCCTGATGAGCATC	
	*SAT*	*Spermidine/spermine N1-acetyltransferase*	SAT_Hi_F1	CTGTGGATGTGACTCAGAAG	173
			SAT_Hi_R1	GCAGATTCTTGCTGATGCGG	
Insulin-like secretion	*AC*	*Adenylyl cyclase*	AC_Hi_F1	GTGTCTTACGTGGCTGAGGC	223
			AC_Hi_R1	CTGCGGTGGGTATAGTCTGC	
	*CCKAR*	*Cholecystokinin A receptor*	CCKAR_Hi_F3	CCCTCCTGATACCTGAAGATG	172
			CCKAR_Hi_R3	GGATTCTCTGGTATTCTTGAC	
	*M3R*	*Muscarinic acetylcholine receptor M3*	M3R_Hi_F1	GGAGTCGATCTCAATGGATC	184
			M3R_Hi_R1	CTAGCAGTGTGGCGATGGAG	
	*PLC*	*Phospholipase C*	PLC_Hi_F1	CTGTGTAGGTATTCACTCGTG	173
			PLC_Hi_R1	CACAGATGAATGAACTGACC	
	*PCLO*	*Piccolo presynaptic cytomatrix protein*	PCLO_Hi_F1	GGCTGGTGATGGACGAAGAC	226
			PCLO_Hi_R1	CCGCGATCTGGAAACGTCAG	
	*SNP25*	*Synaptosome-* *associated protein 25*	SNP25_Hi_F1	GCAGAGCTGAGTGCCGTAGC	232
			SNP25_Hi_R1	GCAACGATCCGAACTACTAC	
	*VAMP3*	*Vesicle-associated membrane protein 3*	VAMP3_Hi_F1	CTAGTGCCAGTGACTGTGAC	198
			VAMP3_Hi_R1	CCACCTCATTCACCTCTCTC	

**Table 3:** *Hippolyte inermis* genes corresponding to human genes in the three functional pathways analyzed, along with the accession number from the shrimp transcriptome [27].

Pathway	Accession Number	Gene	*H. inermis*	Human
Apoptosis	Hippolyte_Body_TRINITY_DN6519_c0_g1	Activating transcription factor	*ATFC*	*ATF4*
	Hippolyte_Body_TRINITY_DN5045_c0_g2	Cathepsin B	*CATB*	*CTSB*
	Hippolyte_Body_TRINITY_DN6311_c0_g4	Cytochrome C	*Cyt-c*	*CYC*
	Hippolyte_Body_TRINITY_DN112258_c0_g1	Death receptor-associated nemesis-like	*Dronc*	*CASP9*
	Hippolyte_Body_TRINITY_DN5290_c0_g1	High-temperature requirement A2 serine peptidase	*HTRA2*	*HTRA2*
	Hippolyte_Body_TRINITY_DN11687_c0_g1	Translocator protein	*TSPO*	*TSPO*
Ferroptosis	Hippolyte_Body_TRINITY_DN4632_c1_g1	Gamma glutamylcysteine synthetase	*GSHI*	*GSX1*
	Hippolyte_Body_TRINITY_DN7730_c5_g1	Glutathione peroxidase 4	*GPX4*	*GPX4*
	Hippolyte_Body_TRINITY_DN28134_c0_g1	Six-transmembrane epithelial antigen of prostate 3	*STEA3*	*STEAP3*
	Hippolyte_Body_TRINITY_DN6660_c0_g1	Spermidine/spermine N1-acetyltransferase	*SAT*	*SAT*
Insulin-like secretion	Hippolyte_Body_TRINITY_DN3141_c0_g1	Adenylyl cyclase	*AC*	*ADCY1*
	Hippolyte_Body_TRINITY_DN83923_c0_g1	Cholecystokinin A receptor	*CCKAR*	*CCKAR*
	Hippolyte_Body_TRINITY_DN80437_c0_g1	Muscarinic acetylcholine receptor M3	*M3R*	*CHRM3*
	Hippolyte_Body_TRINITY_DN22546_c0_g1	Phospholipase C	*PLC*	*PLCL1*
	Hippolyte_Body_TRINITY_DN186639_c0_g1	Piccolo presynaptic cytomatrix protein	*PCLO*	*PCLO*
	Hippolyte_Body_TRINITY_DN5531_c0_g1	Synaptosome-associated protein 25	*SNP25*	*SNAP25*
	Hippolyte_Body_TRINITY_DN21906_c0_g1	Vesicle-associated membrane protein 3	*VAMP3*	*VAMP3*

**Table 4:** Total RNA (ng/μL) purity and integrity (A260/280 and A260/230), with the two extraction methods, for four pools of individuals (1, 2, 3, and 4).

	PL_5_ Replicate	ng/μL	A260/280	A260/230
RNeasy Mini Kit	1	60.9	1.99	0.32
	2	84.5	2.03	0.82
	3	161.6	2.08	0.55
	4	176.8	2.02	0.93
PureLink™ RNA Mini Kit	1	29.9	2.03	1.47
	2	58.4	2.12	0.48
	3	87	2.13	2.07
	4	146.7	2.11	2.07

## Data Availability

Most source data are provided with this paper, in the main text, and in the electronic supplementary material. The transcriptome sequences are stored in a large database, physically located at the Informatics facilities of Ben-Gurion University of the Negev, Israel, and are available on request from the authors.

## Supplementary Materials

The following supporting information can be downloaded at: https://www.mdpi.com/article/10.3390/cimb46060368/s1, Table S1: Name, acronym, and function of the genes analyzed in the present work.
