# Supplementary material for: Molecular Approaches Detect Early Signals of Programmed Cell Death in Hippolyte inermis Leach
Source: Curr Issues Mol Biol. 2024 Jun 18;46(6):6169–85. doi: 10.3390/cimb46060368 (PMC11202572; doi:10.3390/cimb46060368)
Supplement: Supplementary file 1 [file cimb-46-00368-s001.zip › cimb-3043823-supplementary.pdf]

Supplementary Material

Table S1. Name, acronym, and function of the genes analyzed in the present work.

Acronym	Name	Function
Cyt-c	*Cytochrome c*	*Cytochrome c* is an heme protein that is involved in the electron transport chain and plays a role in the regulation of apoptosis.
Atfc	*Activating transcription factor-3*	*Activating transcription factor-3* is a transcription factor that regulates gene expression and is involved in the regulation of cell growth and differentiation.
CatB	*Cathepsin B*	*Cathepsin B* is a lysosomal protease involved in the degradation of cellular proteins.
HtrA2	*High-temperature requirement A2*	*High-temperature requirement A2* is a serine protease that plays a role in the regulation of cell apoptosis and is also involved in the regulation of the unfolded protein response.
Dronc	*Death receptor-associated nemesis-like*	*Death receptor-associated nemesis-like* is an activator of apoptosis and plays a role in the regulation of the cell cycle.
Tspo	*Translocator protein*	*Translocator protein* is a mitochondrial protein involved in the regulation of cellular stress response and the modulation of neurosteroid synthesis.
Pclo	*Parc*	*Parc* is a cytosolic protein involved in the regulation of autophagy and the maintenance of cellular homeostasis.
Ac	*Adenylate Cyclase*	*Adenylate Cyclase* is an enzyme involved in the regulation of intracellular signaling, particularly in the regulation of cAMP signaling.
M3R	*Muscarinic acetylcholine receptor M3*	*Muscarinic acetylcholine receptor M3* is a G protein-coupled receptor involved in the regulation of neurotransmitter signaling, particularly acetylcholine signaling.
Cckar	*Cholecystokinin A Receptor*	The *CCKAR* gene encodes a protein that acts as a receptor for cholecystokinin. The role of *cholecystokinin* signalling in regulating a wide range of physiological processes, including digestion, behaviour, and reproduction.
Vamp3	*Vesicle-associated membrane protein 3*	*Vesicle-associated membrane protein 3* is a SNARE protein involved in the regulation of vesicle trafficking and the fusion of vesicles with the plasma membrane.
Plc	*Phospholipase C*	*Phospholipase C* is an enzyme involved in the regulation of intracellular signaling and the generation of second messengers.
Snp25	*Synaptosomal-associated protein 25*	*Synaptosomal-associated protein 25* is a protein involved in the regulation of vesicle trafficking and the formation of the presynaptic active zone.
Gshi	*Gamma Glutamylcysteine Synthetase*	*Gamma Glutamylcysteine Synthetase* is involved in the metabolism and the maintenance of cellular redox homeostasis.
Stea 3	*Sterile alpha-motif domain-containing protein 3*	*Sterile alpha-motif domain-containing protein 3* is a regulatory protein involved in the regulation of gene expression and the control of cell differentiation.
Gpx4	*Glutathione peroxidase 4*	*Glutathione peroxidase 4* is an enzyme involved in the elimination of reactive oxygen species and the maintenance of cellular redox homeostasis.
Sat	*Spermidine/spermine N1-acetyltransferase*	*Spermidine/spermine N1-acetyltransferase* is an enzyme involved in the regulation of polyamine metabolism and the maintenance of cellular homeostasis.
